# Thermodynamically coupled biosensors for detecting neutralizing antibodies against SARS-CoV-2 variants

**DOI:** 10.1038/s41587-022-01280-8

**Published:** 2022-04-28

**Authors:** Jason Z. Zhang, Hsien-Wei Yeh, Alexandra C. Walls, Basile I. M. Wicky, Kaitlin R. Sprouse, Laura A. VanBlargan, Rebecca Treger, Alfredo Quijano-Rubio, Minh N. Pham, John C. Kraft, Ian C. Haydon, Wei Yang, Michelle DeWitt, John E. Bowen, Cameron M. Chow, Lauren Carter, Rashmi Ravichandran, Mark H. Wener, Lance Stewart, David Veesler, Michael S. Diamond, Alexander L. Greninger, David M. Koelle, David Baker

**Affiliations:** 1 Department of Biochemistry, University of Washington, Seattle, WA USA; 2 Institute for Protein Design, University of Washington, Seattle, WA USA; 3 Department of Medicine, Washington University School of Medicine, St. Louis, MO USA; 4 Department of Laboratory Medicine & Pathology, University of Washington, Seattle, WA USA; 5 Department of Bioengineering, University of Washington, Seattle, WA USA; 6 Howard Hughes Medical Institute, University of Washington, Seattle, WA USA; 7 Department of Pathology & Immunology, Washington University School of Medicine, St. Louis, MO USA; 8 Department of Molecular Microbiology, Washington University School of Medicine, St. Louis, MO USA; 9 Andrew M. and Jane M. Bursky Center for Human Immunology and Immunotherapy Programs, Washington University School of Medicine, Saint Louis, MO USA; 10 Vaccine and Infectious Diseases Division, Fred Hutchinson Cancer Research Center, Seattle, WA USA; 11 Department of Medicine, Division of Allergy and Infectious Diseases, University of Washington, Seattle, WA USA; 12 Translational Research Program, Benaroya Research Institute at Virginia Mason, Seattle, WA USA; 13 Department of Global Health, University of Washington, Seattle, WA USA

**Keywords:** Diagnostic markers, Assay systems, Protein design

## Abstract

We designed a protein biosensor that uses thermodynamic coupling for sensitive and rapid detection of neutralizing antibodies against severe acute respiratory syndrome coronavirus 2 (SARS-CoV-2) variants in serum. The biosensor is a switchable, caged luciferase–receptor-binding domain (RBD) construct that detects serum-antibody interference with the binding of virus RBD to angiotensin-converting enzyme 2 (ACE-2) as a proxy for neutralization. Our coupling approach does not require target modification and can better distinguish sample-to-sample differences in analyte binding affinity and abundance than traditional competition-based assays.

## Abstract (web summary)

SARS-CoV-2 neutralizing antibodies are readily detected with a biosensor.

## Main

With the availability of coronavirus disease 2019 (COVID-19) vaccines, the rise of more transmissible and pathogenic virus mutants^1^ and known time-dependent declines in immunity following infection^2^, there is a need to determine the degree of serological antibody protection from severe acute respiratory syndrome coronavirus 2 (SARS-CoV-2). Knowledge of individual immunity to SARS-CoV-2 is useful not only to determine personal actions but also to guide early therapy of patients and evaluate the efficacy of antibody treatment and vaccines over time against emerging viral variants of concern (VOCs)^3^.

The receptor-binding domain (RBD) of the SARS-CoV-2 spike protein binds to the angiotensin-converting enzyme 2 (ACE-2) receptor on target cells and is the immunodominant target of neutralizing antibodies (nAbs) identified from convalescent and postvaccination plasma^3^. Several SARS-CoV-2 VOCs have exploited this and acquired mutations in the RBD, which allow for escape from nAbs targeting wild-type^4^ (WT; Wuhan-Hu-1 SARS-CoV-2). Serological antibody tests (ideally home-based diagnostics) are critical to evaluate the response to vaccination and viral infection^2^. Assays that measure antibody titer and neutralizing capability exist but are not compatible with home use. Traditional affinity-based immunoassays, such as enzyme-linked immunosorbent assays (ELISAs)^5^, can quantitatively measure antibody titer, but due to inherent complexity and instrumentation, they require a centralized laboratory for diagnostics. Antibody neutralizing capabilities are traditionally measured in cell-based live viral infection assays that require BSL3 facilities^6^. Lateral-flow antigen tests have been introduced, but they are used primarily as binary qualitative tests and report only binding between antibody and antigen rather than neutralization^7^. Recently developed cell-free tools can measure antibody titers but cannot necessarily evaluate neutralization, and none of the currently available tools have estimated neutralization activity against the emerging set of SARS-CoV-2 VOCs^8^. We aimed to develop a sensor technology that can quantitatively measure nAb responses against different isolates of SARS-CoV-2, be adapted for an all-in-solution multiwell format and provide rapid results in 1 hour, which is faster than established ELISA assays measuring SARS-CoV-2 antibody titer (~6 hours) or cell-based neutralization assays (~one to several days).

## Design of the lucCageRBD assay

**Fig. 1:**
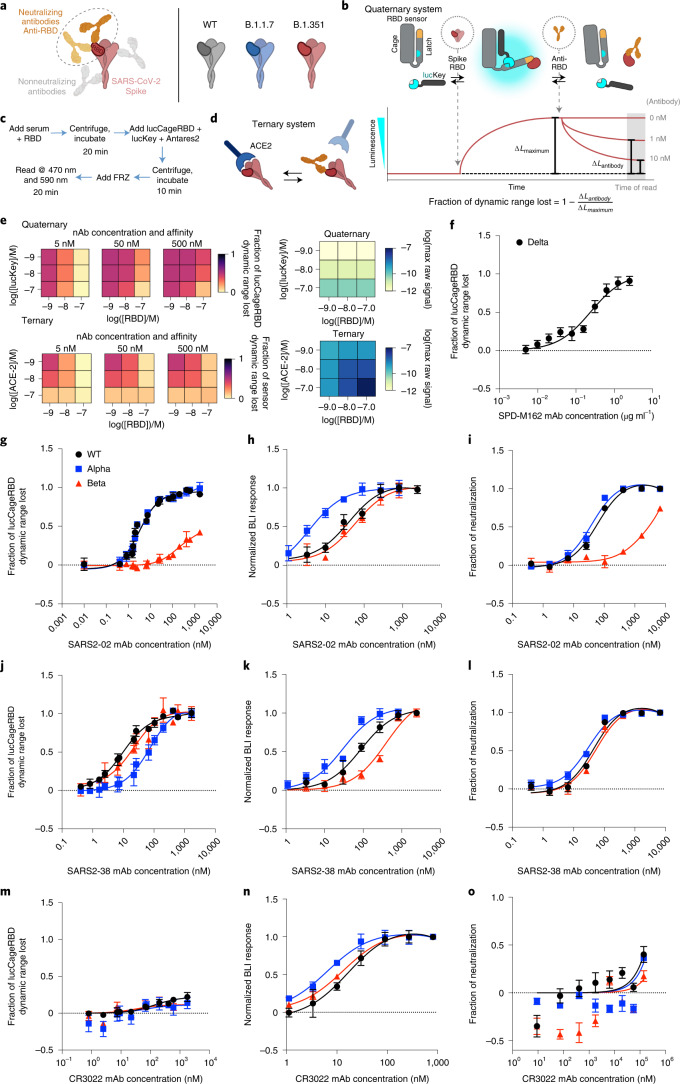
Design and characterization of sensors for mAb detection. **a**, To detect nAbs that primarily block the interaction between ACE-2 and the RBD of SARS-CoV-2 spike WT and other emerging variants, we designed our lucCageRBD assay, which uses the RBD sensor and lucKey. **b**–**d**, Schematic and workflow of the LOCKR nAb biosensor system (quaternary, this work) (**b** and **c**) and ACE-2:RBD outcompetition format (ternary, previous work) (**d**). The RBD sensor contains two domains that interact, the Cage and Latch, the latter of which contains small NanoLuc binary technology (smBiT) of luciferase (blue) and the de novo LCB1 domain (yellow) designed to recognize the ACE-2 binding region of RBD. lucKey contains the Cage-associating key domain and large NanoLuc binary technology (lgBiT) of luciferase (blue). RBD WT or variants bind to LCB1, which together with Key–Cage binding enables reconstitution of luciferase, thus increasing luminescence. nAbs compete for RBD binding, thus shifting for Cage–Latch binding, limiting Key–Cage interaction and disturbing luciferase reconstitution, thus decreasing luminescence. As increasing nAb concentrations should promote decreases in luminescence, we created the fraction of lucCageRBD dynamic range lost metric. **e**, Simulations for the detection and deconvolution of nAb titer from affinity. Each sub-plot represents the sensor’s responses across the different settings of the decision matrix, which is defined by a combination of lucKey and RBD concentrations (quaternary system; left) or ACE-2 and RBD concentrations (ternary system; right). For each sensor system, the raw maximum signal (absence of nAb, used for signal normalization) is also shown (blue heat maps). **f**–**o**, Different concentrations of SPD-M162 (**f**), SARS2-02 (**g**–**i**), SARS2-38 (**j**–**l**) or CR3022 (**m**–**o**) mAbs with 5 nM RBD WT, Alpha, Beta or Delta were tested in the lucCageRBD assay (**f**,**g**,**j** and **m**), BLI for binding to RBD isolates (**h**,**k** and **n**) and spike VOC-containing SARS-CoV-2 live virus (**i** and **l**) or vesicular stomatitis virus (VSV)-based pseudovirus infection (**o**). The lucCageRBD and BLI experiments were performed in triplicate, viral infection experiments were performed in duplicate and data are mean ± s.e.m. Source data

To achieve this goal, we designed an assay that focuses on antibodies competing with RBD:ACE-2 interactions as a proxy for antibody neutralization^8^ (Fig. 1a,b). We adapted a designed coronavirus spike RBD biosensor^9^ consisting of a switchable lucCageRBD protein containing a ‘cage’ domain, which in the closed state of the sensor binds a ‘latch’ domain containing the picomolar affinity RBD binding LCB1 protein^10^, and a lucKey protein that binds to the open state of the sensor, reconstituting luciferase activity^11^. In the absence of RBD, the sensor is in the closed state with the latch bound to the cage, blocking luciferase reconstitution. Upon addition of RBD, the free energy of binding to lucCageRBD, together with that of lucKey, drives switch opening and generation of luminescent signal (Fig. 1b). Because the biosensor is under thermodynamic control and fully reversible, it is capable of detecting RBD-targeted SARS-CoV-2 antibodies that compete with LCB1 at or near the ACE-2 binding interface of RBD. Starting from the open luminescent state of the sensor bound to the RBD, addition of antibody pulls the equilibrium toward the dark closed state (Fig. 1b).

**Extended Data Fig. 1:**
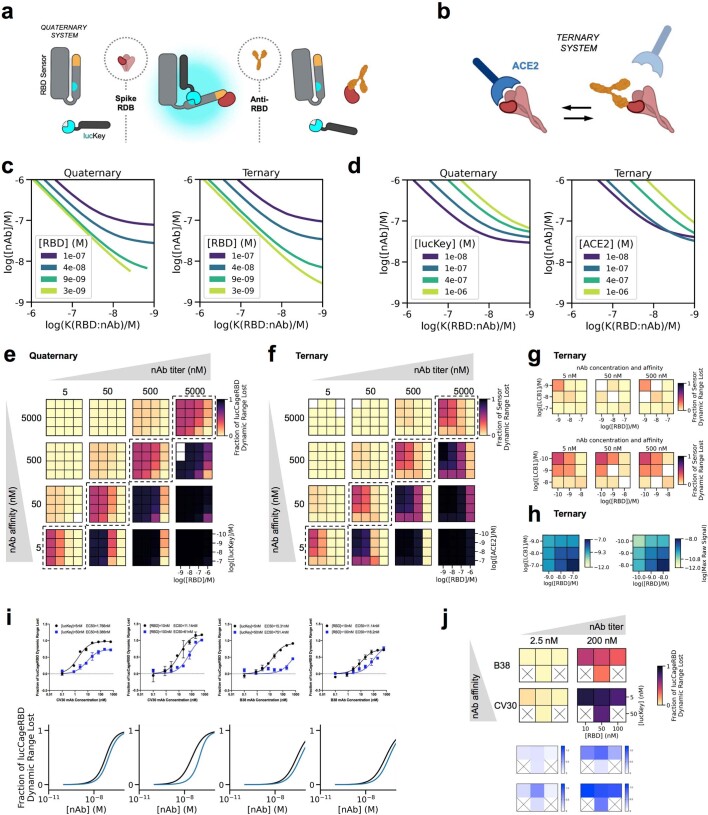
Simulation of biosensor systems and experimental validation. **a**, Schematic of the LOCKR nAb biosensor system (quaternary, this work), and **b**, ACE-2:RBD out-competition format (ternary, previous work). These schematics are the same as in Fig. 1. **c-d**, Simulations of detection capabilities as a function of sensor settings (concentration of sensor components) along the dimension of each independent variable. The decision boundaries (EC50) are shown as solid lines. **c**, The concentration of RBD primarily affects the antibody titer detection limit. For tight antibodies (right-hand side of the plots), the position of the decision boundary (EC50) is solely determined by the concentration of RBD. The concentrations of the untitrated components were: Cage-Latch = 1 nM, lucKey = 50 nM for the quaternary system, and ACE-2 = 1 nM for the ternary system. **d**, Effect of ACE-2 or lucKey concentrations on the ternary and quaternary sensors respectively. Changing lucKey concentration in the quaternary case allows for a better modulation of the nAb affinity detection threshold. The concentration of RBD was set to 50 nM in both cases, and the concentration of Cage-Latch was set to 1 nM for the quaternary system. **e**,**f**, Simulations of outputs for decision matrices of the quaternary (**e**), orternary (**f**) sensors. Each matrix represents the simulated responses for 16 different sensor settings (concentrations of sensor species, indicated at the bottom-right) to a given concentration of nAb of a specific affinity. The quaternary system is capable of deconvoluting affinity and concentration across all combinations that activate the sensor (distinct patterns), while the ternary system returns the same reading for the cases where nAb affinity and concentrations are in the same range (diagonal, highlighted by dashed squares). **g**, Diagonal of decision matrices for ternary systems composed of RBD and LCB1 instead of ACE-2. Sensor readings using the same concentrations of species as the ACE-2:RBD system (top) or adjusted concentrations (bottom). **h**, Simulated maximum raw signals for the ternary system sensor as in (**g**) with unmodified settings (left), and adjusted concentrations (right). The unmodified settings reduce the raw maximum signal range, but also reduce the detection capabilities of the sensor. Adjusting the settings to improve detection also increases the raw maximum signal range. **i-j**, Comparison between simulated and experimental data. **i**, Different concentrations of either CV30 (high affinity; *K*d = 25 nM) or B38 (low affinity; *K*d = 192 nM) mAbs with 1 nM RBD sensor and different concentrations of WT RBD and lucKey in the lucCageRBD assay (experimental and simulation data, top and bottom respectively). **j**, Heat map representation summarizing some of the experimental data from (**i**) for low and high concentrations of CV30 and B38 antibodies (simulation and experimental data, top and bottom respectively). All lucCageRBD experiments were performed in triplicate, representative data are shown, and data are mean ± s.e.m. The quaternary system was simulated with the following parameters: $\Delta G_{open} = 4kcal/mol$, $\left[ {Cage - Latch} \right] = 1nM$, $K\left( {lucKey:oCL} \right) = 5nM$, $K\left( {oCL:RBD} \right) = 500pM$. The ternary system was simulated with $K\left( {ACE2:RBD} \right) = 15nM$, except for **g-h**, where$K\left( {LCB1:RBD} \right) = 500pM$ was used instead. Cases where simulations did not converge are shown as white squares. Source data

Unlike previously described competition assays that directly assess the extent of ACE-2:RBD complex formation (by luciferase reconstitution or capturing of enzyme conjugated to one component) (Fig. 1d), in this thermodynamic coupling scheme, the RBD is unmodified and free in solution. This simplifies testing reactivity against RBD VOCs, because no labeling or further sensory protein engineering is required. A more fundamental advantage, as illustrated by the simulations in Fig. 1e and Extended Data Fig. 1, is that compared to ternary sensor systems that rely on direct competition of the ACE-2–RBD interaction by nAbs, our quaternary sensor system can more readily distinguish analyte binding affinity and abundance, both of which are relevant for diagnosing the success of vaccination. The robustness of the quaternary sensor lies in its tunable sensitivity to detect the abundance/dissociation constant (*K*_d_) of unknown antibodies in serum or plasma samples and is the first step of the lucCageRBD assay setup. This step requires pretesting different configurations or evaluating simultaneously several configurations to determine the fraction decrease using different dynamic ranges (as described in the decision matrix in Fig. 1e). Distinguishing signals due to higher concentrations of more weakly binding analytes from those due to lower concentrations of more strongly binding analytes is challenging for the simple outcompetition system, because disruption of the ACE-2:RBD complex (and hence detection) is affected by the concentration and affinity of the competitor to the same proportional extent. Taking measurements for different configurations of the quaternary sensor system (changing the concentrations of the RBD and lucKey sensor components) generates decision matrices (Fig. 1e) that better discriminate analyte concentration and affinity across a broad range of values, including the constant [concentration]/*K*_d_ regime that cannot be discriminated by the ternary sensor (sensor activation across the full analyte affinity and concentration ranges are shown in Extended Data Fig. 1). Another advantage of our quaternary system is that the maximum raw sensor response in the absence of nAb (used for signal normalization; decision matrices at the far right in Fig. 1e) varies less between the different sensor configurations than in the ternary system, because the concentration of the limiting component for signal generation (lucCageRBD) is constant; variation in maximum signal is a problem for luminescent (or other enzyme coupled) sensors because of substrate depletion effects and instrument detection limits. This variation in maximum signal can be reduced in the ternary system if one component is kept fixed; however, in this case, the high-affinity/low-concentration and low-affinity/high-concentration regimes are even harder to resolve (Extended Data Fig. 1g,h). Substituting ACE-2 for a higher-affinity RBD binder like LCB1 does not alleviate this problem. Finally, our quaternary system is considerably more engineerable. The affinities of the Latch and Key for the Cage can both be tuned to maximize the dynamic range of the system for the relevant analyte affinities and concentrations, whereas to tune response in the ternary system, only mutations at the interface between the interacting partners can be made, which may be insufficient to obtain the desired detection range.

## LucCageRBD assay estimates neutralization potency of mAbs

**Extended Data Fig. 2:**
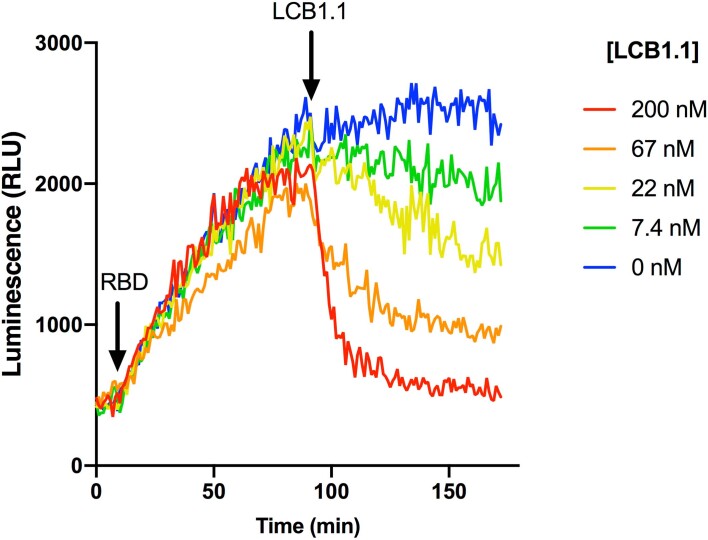
Reversibility of lucCageRBD. Time-course luminescence of the lucCageRBD assay using 200 nM of RBD WT and different concentrations of the *de novo* LCB1.1 binder. All experiments were performed in triplicate, representative data are shown. Source data

**Extended Data Fig. 3:**
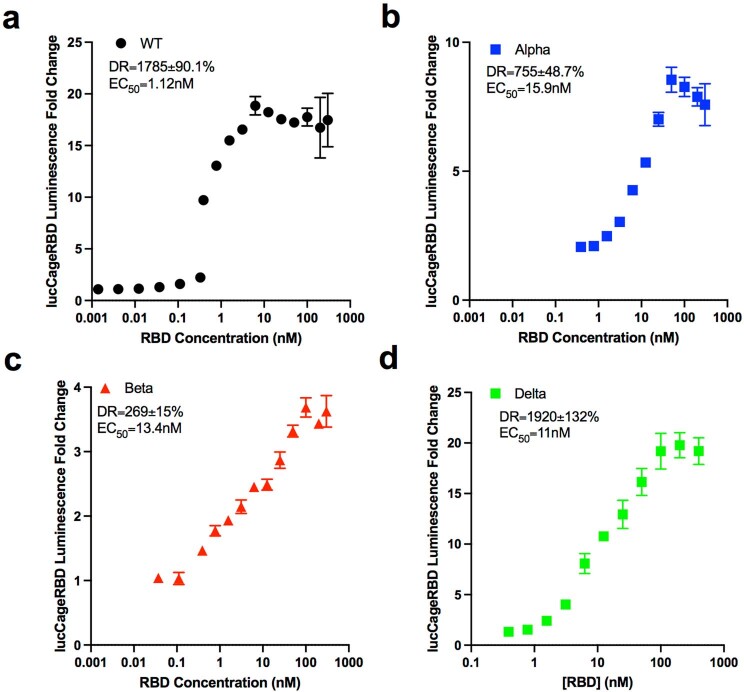
Detection of RBD strains by lucCageRBD. **a–d**, Fold change increase in luminescence from different concentrations of RBD WT (**a**), Alpha (**b**), Beta (**c**), Delta (**d**) tested in the lucCageRBD assay. All experiments were performed in triplicates and data are mean ± s.e.m. Source data

**Extended Data Fig. 4:**
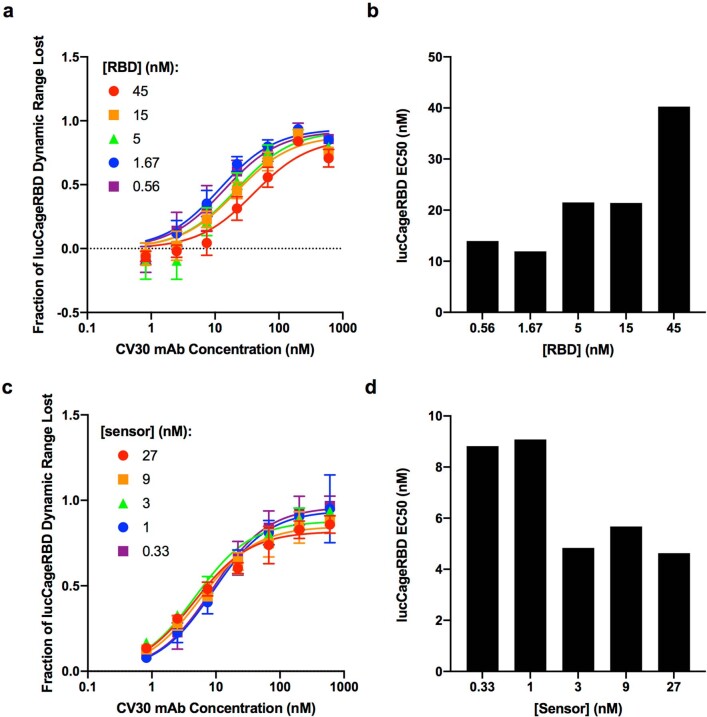
Effects of RBD and sensor titration on detecting SARS-CoV-2 antibodies. **a**,**b**, Resulting fraction of lucCageRBD dynamic range lost (**a**) and lucCageRBD EC50 (**b**) from different concentrations of mAb CV30 with different concentrations of RBD WT in 1 nM of RBD sensor and lucKey. **c-d**, Resulting fraction of lucCageRBD dynamic range lost (**c**) and lucCageRBD EC50 (**d**) from different concentrations of mAb CV30 with different concentrations of RBD sensor and lucKey (1:1 stoichiometry maintained) and with 5 nM RBD. All experiments were performed in triplicates and data are mean ± s.e.m. Source data

To characterize the quaternary sensor system experimentally, we investigated the modulation of lucCageRBD signal by combinations of RBD (and RBD variants) and RBD binding proteins. Addition of 333 pM RBD to the sensor resulted in a rapid (*t*_1/2_ = 22 min) fivefold increase in luminescence from baseline, which was rapidly quenched (*t*_1/2_ = 10 min) by subsequent addition of 200 nM LCB1, which competitively inhibits RBD binding to the RBD sensor (Extended Data Fig. 2). LucCageRBD also detects Alpha, Beta and Delta RBDs (Extended Data Fig. 3) and the interaction of these proteins with nAbs, such as the Delta-nAb SPD-M162 (Fig. 1f), a validated anti-SARS-CoV-2 spike RBD IgM. To quantify the extent of binding of a competitor antibody or other RBD binding protein, we used as a metric the fraction decrease in total sensor dynamic range (Fig. 1b; this is also used in the simulations above). As predicted by the model, half-maximal effective concentration (EC_50_) values for lucCageRBD detection of the neutralizing mAb CV30 (ref. ^12^) increase with increasing RBD concentration and decrease with increasing sensor concentration (maintaining 1:1 stoichiometry with RBD) (Extended Data Fig. 4), illustrating how changing RBD and sensor concentrations can tune the sensitivity of the lucCageRBD assay.

**Extended Data Fig. 5:**
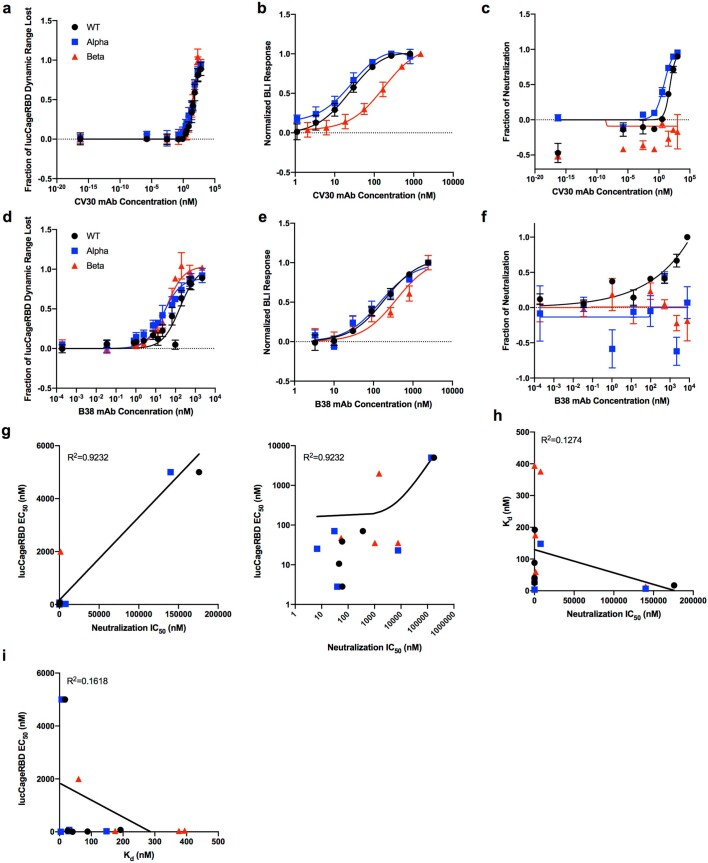
Monoclonal antibodies tested for lucCageRBD, binding, and neutralization. **a–f**, Different concentrations of either CV30 (**a-c**) or B38 (**e-g**) mAbs with 5 nM RBD WT, Alpha, and Beta were tested in the lucCageRBD assay (**a, d**), BLI for binding to RBD strains (**b, e**), and spike VoC-presenting pseudovirus infection (VSV-based for **c**, HIV-based for **f**). Comparison of lucCageRBD EC50 and neutralization IC50 plotted linear-linear (left) or log-log (right) (**g**), binding affinity (k_d_) and neutralization IC50 (**h**), and lucCageRBD EC50 and k_d_ (**i**). All lucCageRBD and BLI experiments were performed in triplicate, neutralization experiments were performed in at least duplicate and data are mean ± s.e.m. Source data

To evaluate the detection of nAbs through equilibrium perturbation of the lucCageRBD:RBD system, we compared binding to the spike, virus neutralization (live virus for Fig. 1i,l, pseudovirus for Fig. 1o and Extended Data Fig. 5c,f), and sensor activation over a set of five anti-spike mAbs (SARS2-02, SARS2-38, CV30, B38 and CR3022)^13–16^ for the WT, Alpha, and Beta spike variants (Fig. 1g–o and Extended Data Fig. 5), one of which (CR3022) does not interfere with ACE-2–RBD interaction^15^ and accordingly has little effect in the lucCageRBD assay (Fig. 1m). Over this set of antibodies and spike variants, virus neutralization correlates with sensor activation better than with spike binding affinity (Extended Data Fig. 5g–i), as expected, because the sensor is only sensitive to binding near the ACE-2 binding site which is the target of most nAbs. As an example, the SARS2-02 antibody binds (by biolayer interferometry (BLI)) Beta and WT RBD (Fig. 1h) with roughly equal affinities, but neutralizes infectious SARS-CoV-2 (live virus) containing WT and Alpha spike proteins much more potently (20- to 40-fold increase in half-maximum inhibitory concentration (IC_50_)) than Beta spike-containing virus^13^ (Fig. 1i). Consistent with the neutralization results, the SARS2-02 antibody produces a large decrease in lucCageRBD signal with WT and Alpha RBD but a partial response with Beta RBD (EC_50_ ~40-fold increased) (Fig. 1g and Supplementary Table 1). To confirm the ability to differentiate nAb concentration and affinity suggested by the simulations in Fig. 1e, we assayed two antibodies (CV30 and B38) with different affinities for WT RBD at two different concentrations each, using four different sensor settings, and found that the differential sensor readings for each condition were consistent with the computational model (Extended Data Fig. 1i,j).

## LucCageRBD assay robustly detects nAbs in complex samples, including clinical samples

**Fig. 2:**
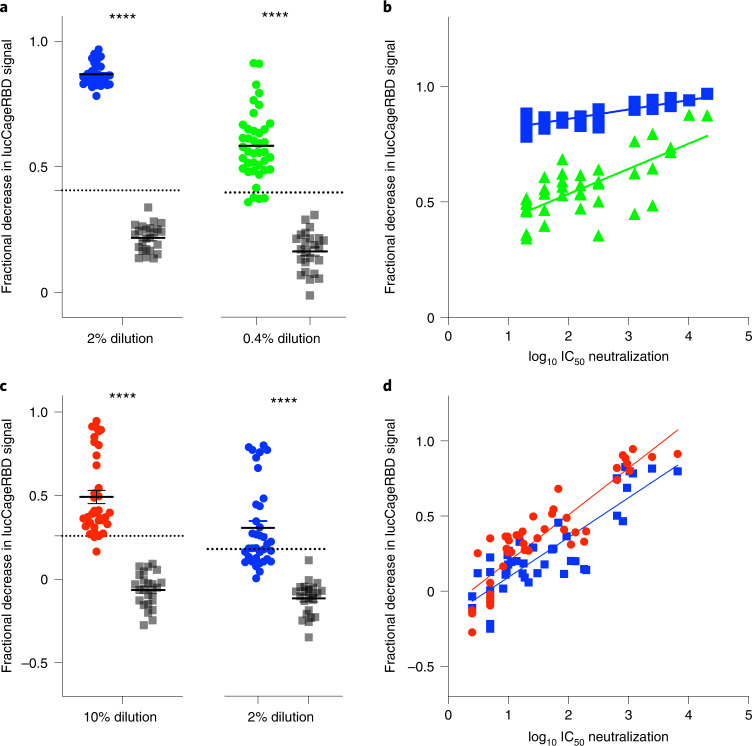
Detection of nAbs in clinical samples. **a**–**d**, Serum from convalescent or vaccinated patients (positive samples, *n* = 40 for WT, *n* = 36 for Delta) and pre-2019 samples (negative samples, *n* = 24 for both WT and Delta) were collected and tested in the lucCageRBD assay at the indicated dilutions (**a** and **c**) and pseudovirus infection (**b** and **d**) against both WT and Delta RBD/spike. **a**, Associated sensitivity, specificity, positive predictive value and negative predictive value statistics are in Supplementary Table 2 and were calculated with 3 s.d. above negative sample mean (dotted line in **a** and **c**) as the predictive cutoff. **b**, 2% dilution, *R*^2^ = 0.686, Pearson’s *r* = 0.828; 0.4% dilution, *R*^2^ = 0.522, Pearson’s *r* = 0.722. **d**, 10% dilution, *R*^2^=0.807, Pearson’s *r* = 0.898; 2% dilution, *R*^2^ = 0.755, Pearson’s *r* = 0.869, ****P < 0.0001, two-tailed Student’s *t* test. **a**, 2% dilution, ****P = 3.84 × 10^−38^; 0.4% dilution, 5.5 × 10^−23^. **c**, 10% dilution, ****P = 4.28 × 10^−17^; 2% dilution, ****P = 3.21 × 10^−12^. Viral infection experiments were performed in duplicate and data are mean ± s.e.m. Source data

**Extended Data Fig. 6:**
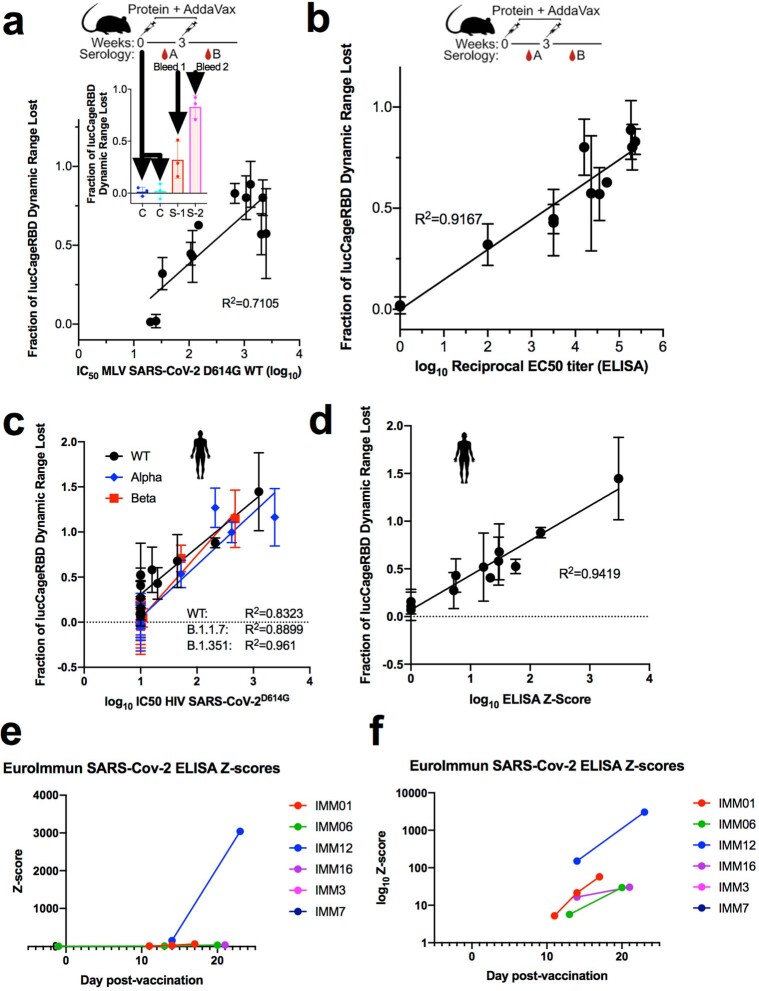
Detection of neutralizing antibodies in vaccinated serum. **a**,**b**, Serum (10%) from vaccinated mice were collected and tested for pseudovirus infection (**a**), in ELISA (log_10_ reciprocal EC50 titer) (**b**), and in the lucCageRBD assay (**a, b**) (n=12 serum samples). **(**Inset) Mouse serum samples pre-immunization, post-prime dosing (week 3), and post-boost dosing (week 5) were collected and tested in the lucCageRBD assay. **c-d**, Serum (10%) from vaccinated patients were tested for pseudovirus infection (**c**), in ELISA (log_10_ reciprocal EC50 titer) (**d**), and in the lucCageRBD assay (**c, d**) using RBD WT, Alpha, and Beta (n=12 serum samples). **e-f**, Serum collected from patients pre and post vaccination were tested in the EuroImmun ELISA for anti-spike antibodies. Patient’s anti-spike antibody levels (measured by Z-score) were measured several days after vaccination. Z-score is plotted either in linear (**e**) or log_10_ scale (**f**). All neutralization experiments were performed in at least duplicates, all lucCageRBD and ELISA experiments were performed in triplicate and data are mean ± s.e.m. Source data

**Extended Data Fig. 7:**
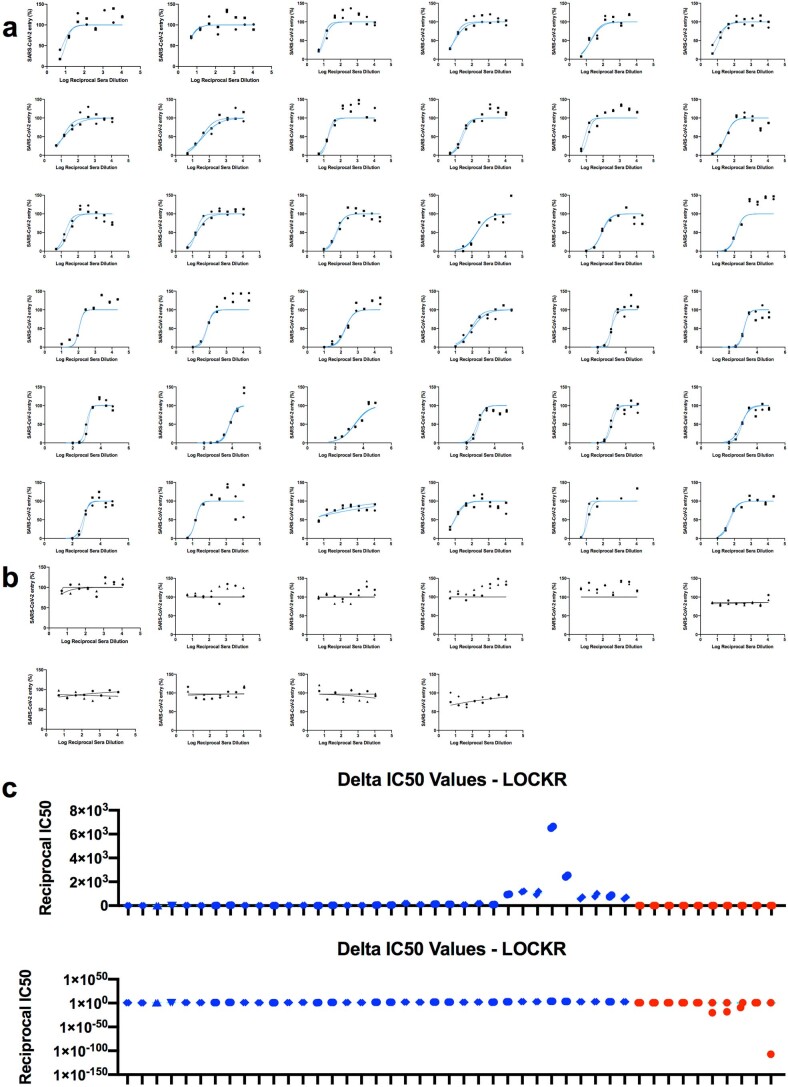
Pseudovirus neutralization curves for clinical samples. **a,b**, (**a**) Serum from convalescent and/or vaccinated patients and pre-2019 serum samples (**b**) were collected and tested for pseudovirus infection against Delta spike. **c**, IC50 values of serum from convalescent and/or vaccinated patients (blue) or pre-2019 serum samles (red) for blocking Delta-spike containing pseudoviral entry. Bar graph is plotted either linearly (top) or logarithmically (bottom). Source data

**Extended Data Fig. 8:**
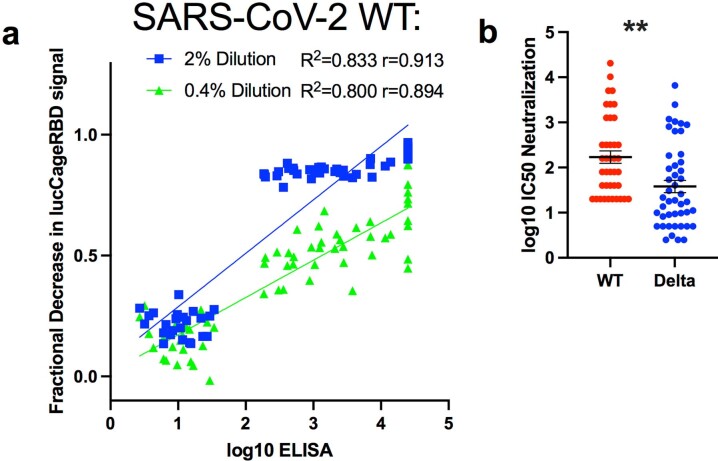
Detection of neutralizing antibodies in clinical samples. **a**, Serum from convalescent and/or vaccinated patients (positive samples, n=40) and pre-2019 samples (negative samples, n=24) were collected and tested in both the lucCageRBD and ELISA assay and pseudovirus infection against WT RBD. **b**, The same samples were tested for neutralization of either WT or Delta spike-presenting pseudovirus with log10 IC50 neutralization reported. Viral infection experiments were performed in duplicate, and data are mean ± s.e.m. r represents pearson’s coefficient. **P=0.0012, two-tailed student’s t-test. Source data

We next investigated whether the correlation between sensor response and neutralizing activity observed over the panel of monoclonal antibodies (mAbs) held for polyclonal antibodies in serum. As complex biological matrices can affect absolute luminescent readings, we used Antares2 as a BRET reference for internal calibration, and used as a measure of sensor activation the ratio of luminescence signal to the internal standard^11^ (Methods). Before vaccination, mouse serum samples^17^ did not decrease activation, whereas serum samples after prime dosing (week 3) and after boost dosing (week 5) showed progressively larger decreases in activation. Decreases in the luminescence ratio correlate (*R*^2^ = 0.711) with the log_10_ IC_50_ values against WT spike-presenting pseudovirus (Extended Data Fig. 6a) and the log_10_ reciprocal EC_50_ titer measured in ELISA (*R*^2^ = 0.917) (Extended Data Fig. 6b). In serum samples from humans vaccinated with BNT162b2 against WT, Alpha and Beta RBD, the lucCageRBD loss in dynamic range correlates with the SARS-CoV-2 (WT) antibody titer detected from ELISA using the log_10_ of the *Z*-score metric (Extended Data Fig. 6d) (*R*^2^ = 0.942) and with log_10_ IC_50_ values against pseudovirus displaying either WT (*R*^2^ = 0.832), Alpha (*R*^2^ = 0.89) or Beta spike (*R*^2^ = 0.961) proteins (Extended Data Fig. 6c). We next evaluated lucCageRBD responses over 40 samples containing SARS-CoV-2 nAbs from persons convalescent, vaccinated or both with a broad range of titers and 24 pre-2019 samples against different SARS-CoV-2 VOCs and dilutions (Fig. 2 and Supplementary Table 2). For both WT and Delta RBD, the lucCageRBD assay positively correlates with the SARS-CoV-2 antibody titer detected from cell-based pseudovirus neutralization experiments (Fig. 2b,d and Extended Data Fig. 7) and ELISA (Extended Data Fig. 8), and the discrimination between pre- and post-COVID exposure was nearly perfect for both WT and Delta versions of the sensor (Fig. 2a,c), with weaker potency generally observed against Delta. These results suggest that our assay can serve as a proxy for a much more involved virus neutralization assay against WT and VOC viruses.

## Discussion

**Extended Data Fig. 9:**
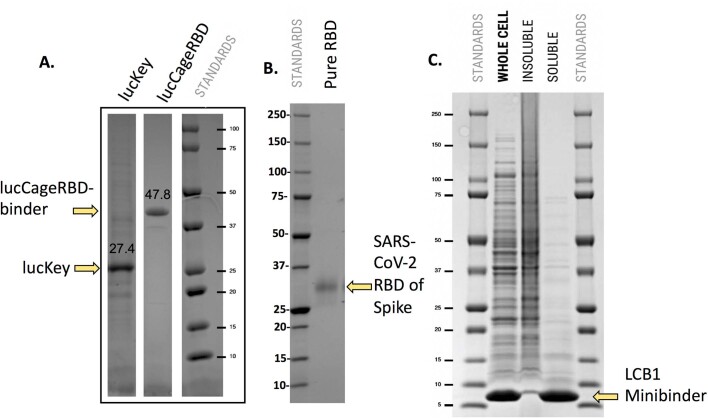
Production and purification of recombinant proteins used in LOCKR biosensor for serological diagnosis of neutralizing antibodies. **a**, Individual His6 tagged versions of lucKey (27.4 KDa) and lucCageRBD (47.8 KDa) are expressed in *E. coli* and following cell lysis are purified by IMAC using 50 mM imidazole in buffer elution. **b**, His6 tagged SARS-CoV-2 RBD (30 KDa) is expressed as a secreted protein in human 293 cells transiently transfected with DNA vectors encoding the protein which is purified from serum free culture medium by IMAC using 50 mM imidazole in buffer elution. **c**, His6 tagged LCB1 anti-SARS-CoV-2 RBD minibinder (7 KDa) is expressed in *E. coli* (whole cell). Following cell lysis by heating to 95 °C and centrifugation to separate insoluble from soluble material, the LCB1 minibinder protein is >90% pure. Samples shown in A-C were analyzed by SDS-PAGE with reducing agent and Coomassie blue staining (molecular weight standards shown). All experiments were performed in triplicate, representative data are shown. Source data

**Extended Data Fig. 10:**
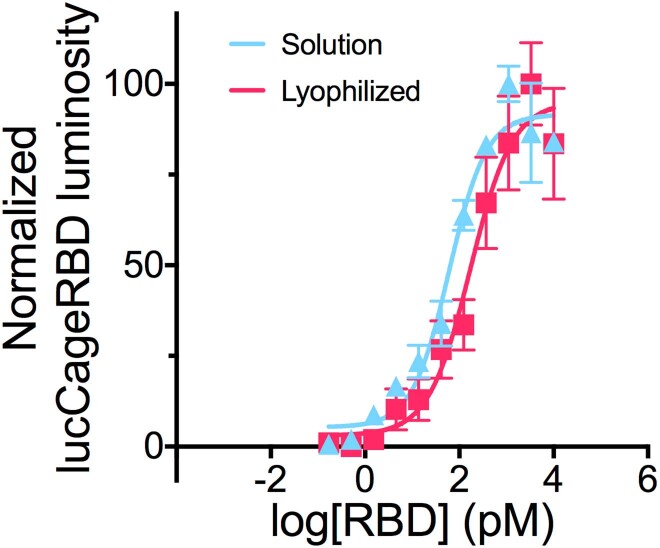
lucCageRBD biosensor sensitivity before and after lyophilization. lucCageRBD and lucKey were lyophilized at 10X concentration in a 96-well plate. After reconstitution in liquid format, the biosensor was tested at a final concentration of 1 nM lucCageRBD and 1 nM lucKey for the detection of serially diluted RBD. All experiments were performed in triplicates and data are mean ± s.e.m. Source data

Our sensor complements previously described COVID-19 serological tests. First, our sensor does not require labeling of the RBD or variant RBDs, which makes it straightforward to substitute in new escape variant RBDs as they are identified. Previous studies have demonstrated detection of antibodies against the WT RBD; here, we demonstrate differentiation between antibodies based on their extent of reaction with WT and escape variant RBDs. Second, the components of the sensor can be readily made in *Escherichia coli* and can be lyophilized without loss of performance (Extended Data Figs. 9 and 10); hence, there are potential advantages in shelf life and manufacturing. The low stability of the ACE-2 protein has complicated high throughput, one-step serological detection of nAbs^18^, and use of the hyperstable LCB1 instead avoids this problem. A potential limitation of the lucCageRBD assay is that it detects only antibodies which bind at the ACE-2 binding site of the RBD and hence cannot quantify antibodies binding to other regions of spike or nucleocapsid^19^, but the RBD is a dominant target of nAbs, and hence, as confirmed by the strong correlation with neutralization titer over the human samples, this is not a substantial limitation. Further research will focus on incorporating the sensor into a scalable 384-well high-throughput format or a low-cost point-of-care diagnostic testing platform. More generally, with the recent advances of computational design to generate high-affinity binding proteins and protein switches, the approach described in this paper should be readily extensible to quantification of the binding affinity and abundance of a wide variety of analytes of interest.

## Methods

### Cells

HEK293T/17 is a human embryonic kidney cell line (ATCC, CRL-11268). The HEK-ACE-2 adherent cell line was obtained through BEI Resources (NR-52511). All adherent cells were cultured at 37 °C with 8% CO_2_ in flasks with Dulbecco’s modified Eagle medium (DMEM) supplemented with 10% fetal bovine serum (FBS) (Hyclone) and 1% penicillin-streptomycin (PenStrep).

HEK293F is a female human embryonic kidney cell line transformed and adapted to grow in suspension (Life Technologies). HEK293F cells were grown in 293FreeStyle expression medium (Life Technologies), cultured at 37 °C with 8% CO_2_ and shaking at 130 rpm. Expi293F cells are derived from the HEK293F cell line (Life Technologies). Expi293F cells were grown in Expi293 Expression Medium (Life Technologies), cultured at 36.5 °C with 8% CO_2_ and shaking at 150 rpm.

Vero E6 (CRL-1586, American Type Culture Collection), Vero-TMPRSS2 (a gift of S. Ding, Washington University) and Vero-hACE2-TMPRSS2 (a gift of A. Creanga and B. Graham, National Institutes of Health (NIH)) cells were cultured at 37 °C in DMEM supplemented with 10% FBS, 10 mM HEPES (pH 7.3), 1 mM sodium pyruvate, 1× nonessential amino acids and 100 U ml^−1^ PenStrep. Vero-TMPRSS2 cell cultures were supplemented with 5 μg ml^−1^ blasticidin. TMPRSS2 expression was confirmed using an anti-V5 antibody (Thermo Fisher Scientific, 2F11F7) or anti-TMPRSS2 mAb (Abnova, Clone 2F4) and APC-conjugated goat anti-mouse IgG (BioLegend, 405308). Vero-hACE2-TMPRSS2 cell cultures were supplemented with 10 μg ml^−1^ puromycin.

### mAbs

The murine mAbs SARS2-02 and SARS2-38 studied were isolated from BALB/c mice immunized with SARS-CoV-2 spike and RBD proteins and have been described previously^17^. Genes encoding CR3022, B38 and CV30 heavy and light chains were ordered from GenScript and cloned into pCMV/R. Antibodies were expressed by transient cotransfection of both heavy- and light-chain plasmids in Expi293F cells using PEI-MAX (Polyscience) transfection reagent. Cell supernatants were harvested and clarified after 3 or 6 days, and protein was purified using protein A chromatography (Cytiva). SPD-M162 was obtained from AcroBiosystems (AM122).

### Live virus production

The 2019n-CoV/USA_WA1/2020 isolate of SARS-CoV-2 was obtained from the US Centers for Disease Control. The Alpha isolate was obtained from a nasopharyngeal sample after propagation on Vero-hACE2-TMPRSS2 cells^3^. The chimeric WA1/2020 displaying Beta virus has been described previously^3^. All viruses were deep-sequenced and tittered on Vero-TMPRSS2 cells, and experiments were performed in an approved biosafety level 3 facility.

### Pseudovirus production

Murine leukemia virus (MLV)-based and human immunodeficiency virus (HIV) SARS-CoV-2 S pseudotypes were prepared as previously described^20,21^. Briefly for MLV, HEK293T cells were cotransfected using Lipofectamine 2000 (Life Technologies) with an S-encoding plasmid, an MLV Gag–Pol packaging construct and the MLV transfer vector encoding a luciferase reporter according to the manufacturer’s instructions. For HIV, HEK293T cells were cotransfected using Lipofectamine 2000 (Life Technologies) with an S-encoding plasmid, an HIV Gag-Pol, Tat, Rev1B packaging construct and the HIV transfer vector encoding a luciferase reporter according to the manufacturer’s instructions. Cells were washed three times with Opti-MEM before transfection and incubated for ~5 h at 37 °C with transfection medium. DMEM containing 10% FBS was added for ~60 h. The supernatants were harvested by spinning at 2,500 *g*, filtered through a 0.45-mm filter, concentrated with a 100-kDa membrane for 10 min at 2,500 *g* and then aliquoted and stored at −80 °C before use.

D614G SARS-CoV-2 S (YP 009724390.1), Alpha S, Beta S and Delta S pseudotypes VSVs were prepared as described previously^22^. Briefly, 293T cells in DMEM supplemented with 10% FBS, 1% PenStrep seeded in 10-cm dishes were transfected with the plasmid encoding for the corresponding S glycoprotein using Lipofectamine 2000 (Life Technologies) following the manufacturer’s indications. One day after transfection, cells were infected with VSV(G*ΔG-luciferase) and after 2 h were washed five times with DMEM before adding medium supplemented with anti-VSV-G antibody (ATCC, I1- mouse hybridoma supernatant, CRL- 2700). Virus pseudotypes were harvested 18–24 h after inoculation, clarified by centrifugation at 2,500 *g* for 5 min, filtered through a 0.45-μm cutoff membrane, concentrated 10 times with a 30-kDa cutoff membrane, aliquoted and stored at −80 °C.

### Mouse serum

Female BALB/c (stock 000651) mice were purchased at the age of 4 weeks from The Jackson Laboratory and maintained at the Comparative Medicine Facility at the University of Washington (Seattle, WA), accredited by the American Association for the Accreditation of Laboratory Animal Care International. At 6 weeks of age, mice were immunized, and 3 weeks later, animals were boosted. Before inoculation, immunogen suspensions were gently mixed 1:1 vol/vol with AddaVax adjuvant (Invivogen) to reach a final concentration of 0.009 or 0.05 mg ml^−1^ antigen. Mice were injected intramuscularly into the gastrocnemius muscle of each hind leg using a 27-G needle (BD) with 50 ml per injection site (100 ml total) of immunogen under isoflurane anesthesia. To obtain sera, all mice were bled 2 weeks after prime and boost immunizations. Blood was collected via submental venous puncture and rested in 1.5-ml plastic Eppendorf tubes at room temperature for 30 min to allow for coagulation. Serum was separated from hematocrit via centrifugation at 2,000 *g* for 10 min. Complement factors and pathogens in isolated serum were heat-inactivated via incubation at 56 °C for 60 min. Serum was stored at 4 °C or −80 °C until use. Mouse sera in this study were used in a previous study^17^.

### Human serum and ELISA

Immune human sera/plasma specimens were from persons with documented SARS-CoV-2 infection in the United States between February and August 2020. Samples were obtained before vaccination or after vaccination with BNT162b2 or M1273 mRNA, after obtaining informed written consent in an institutional review board-approved protocol^23^. Antibodies to the SARS-CoV-2 spike protein were measured using an ELISA specific for anti-S1 IgG (Euroimmun). Antibody levels were quantified by conversion of the optical density to a *z*-score relative to prepandemic serum anti-S1 IgG concentrations, as previously described^23^. Sera tests were serial specimens obtained following immunization of laboratory volunteers in an institutional review board-compliant protocol. The negative samples (no SARS-CoV-2 nAbs) were serum samples collected for VZV research in 2016 and 2017, before SARS-CoV-2 was spreading in the United States.

### General procedures for bacterial protein production and purification

The *E. coli* Lemo21(DE3) strain (New England Biolabs) was transformed with a pET29b^+^ plasmid encoding the synthesized gene of interest. Cells were grown for 24 h in LB medium supplemented with kanamycin. Cells were inoculated at a 1:50 ml ratio in the Studier TBM-5052 autoinduction medium supplemented with kanamycin, grown at 37 °C for 2–4 h and then grown at 18 °C for an additional 18 h. Cells were collected by centrifugation at 4,000 *g* at 4 °C for 15 min and resuspended in 30 ml lysis buffer (20 mM Tris-HCl, pH 8.0, 300 mM NaCl, 30 mM imidazole, 1 mM PMSF and 0.02 mg ml^−1^ DNase). Cell resuspensions were lysed by sonication for 2.5 min (5 s cycles). Lysates were clarified by centrifugation at 24,000 *g* at 4 °C for 20 min and passed through 2-ml Ni-NTA nickel resin (Qiagen, 30250) pre-equilibrated with wash buffer (20 mM Tris-HCl, pH 8.0, 300 mM NaCl and 30 mM imidazole). The resin was washed twice with 10 column volumes (CVs) of wash buffer, and then eluted with 3 CVs elution buffer (20 mM Tris-HCl, pH 8.0, 300 mM NaCl and 300 mM imidazole). The eluted proteins were concentrated using Ultra-15 Centrifugal Filter Units (Amicon) and further purified by using a Superdex 75 Increase 10/300 GL (GE Healthcare) size exclusion column in TBS (25 mM Tris-HCl, pH 8.0, and 150 mM NaCl). Fractions containing monomeric protein were pooled, concentrated and snap-frozen in liquid nitrogen and stored at −80 °C.

### Plasmid construction for RBD

The SARS-CoV-2 RBD (UniProt: P0DTC2) (BEI NR-52422), Alpha (N501Y), Beta (K417N, E484K and N501Y) and Delta (L452R and T478K) constructs were synthesized by GenScript into pcDNA3.1- or CMVR with an N-terminal mu-phosphatase signal peptide and a C-terminal octa-histidine tag (GHHHHHHHH). The boundaries of the construct are N-328RFPN331 and 528KKST531-C (ref. ^17^).

### Transient transfection

RBD proteins were produced in Expi293F cells grown in suspension using Expi293F expression medium (Life Technologies) at 33 °C, 70% humidity, 8% CO_2_ rotating at 150 rpm. The cultures were transfected using PEI-MAX (Polyscience) with cells grown to a density of 3.0 million cells per milliliter and cultivated for 3 days. Supernatants were clarified by centrifugation (5 min at 4,000 rcf), addition of polydiallyldimethylammonium chloride solution to a final concentration of 0.0375% (Sigma Aldrich, 409014) and a second spin (5 min at 4,000 rcf).

### Purification of RBD

His-tagged RBD was purified from clarified supernatants via a batch bind method, where each clarified supernatant was supplemented with 1 M Tris-HCl, pH 8.0, to a final concentration of 45 mM and 5 M NaCl to a final concentration of 310 mM. Talon cobalt affinity resin (Takara Bio) was added to the treated supernatants and allowed to incubate for 15 min with gentle shaking. Resin was collected using vacuum filtration with a 0.2-mm filter and transferred to a gravity column. The resin was washed with 20 mM Tris, pH 8.0, 300 mM NaCl, and the protein was eluted with 3 CVs of 20 mM Tris, pH 8.0, 300 mM NaCl and 300 mM imidazole. The batch bind process was then repeated and the first and second elutions combined. SDS-PAGE was used to assess purity. Following immobilized metal affinity chromatography purification, the elution was concentrated and applied to a Cytiva S200 Increase column equilibrated with 20 mM Tris 150 mM NaCl, pH 8.0, and the peak of interest was collected and quantified using A280. The purified RBD was qualified using BLI to confirm binding using CR3022 and hACE2-Fc.

### In vitro bioluminescence characterization with mAbs

A Synergy Neo2 Microplate Reader (BioTek) was used for all in vitro bioluminescence measurements. Assays were performed in 50% HBS-EP (GE Healthcare Life Sciences) plus 50% Nano-Glo assay buffer. For each well of a white opaque 96-well plate, 5 μl of 10× lucCage, 5 μl of 10× lucKey, 5 μl of 10× RBD and 5 μl of 10× antibody and remaining volume of buffer for in total 50 μl were mixed to reach the indicated concentration and ratio. The lucCage and lucKey components were incubated for 30 min at room temperature to enable pre-equilibration. The plate was centrifuged at 1,000 *g* for 10 s and incubated at room temperature for a further 30 min. Then, 15 μl of 100× diluted furimazine (Promega, Nano-Glo luciferase assay reagent) was added to each well. Bioluminescence measurements in the absence of target were taken every 1 min (0.1 s integration and 10 s shaking during intervals) for a total of 30 min. To calculate the percent decrease in dynamic range for the graphs, the following formula was used:

$${\mathrm{Fraction}}\,{\mathrm{of}}\,{\mathrm{lucCageRBD}}\,{\mathrm{dynamic}}\,{\mathrm{range}}\,{\mathrm{lost}} = 1 - \frac{{L_x - L_{\mathrm{min}}}}{{L_{\mathrm{max}} - L_{\mathrm{min}}}},$$

where *L*_*x*_ is the luminosity with 5 nM RBD and the tested antibody concentration, *L*_min_ is the luminosity when no RBD is added, and *L*_max_ is the luminosity when only 5 nM RBD is added. To derive EC_50_ values from the bioluminescence-to-analyte plot, the top three peak bioluminescence intensities at individual analyte concentrations were averaged, subtracted from blank and used to fit the sigmoidal 4PL curve.

### Detection of spiked RBD in human serum specimens

Serum specimens were derived from excess plasma or sera from adults (>18 years) of both genders provided by the Director of the Clinical Chemistry Division, the hospital of University Washington. Serum specimens were obtained in compliance with approval by the University of Washington Human Subjects Division. All anonymized donor specimens were provided deidentified. Because the donors consented to have their excess specimens be used for other experimental studies, they could be transferred to our study without additional consent. All samples were passed through 0.22-μm filters before use, and 5 μl of 10× monomeric RBD (10 or 1,000 nM), 5 μl of 10× lucCage (10 nM), 5 μl of 10× lucKey (10 nM), 5 μl of 10× Antares2 (0.5 nM) and the indicated amount of human donor serum or simulated nasal matrix were mixed with 1:1 HBS/Nano-Glo assay buffer to reach a total volume of 50 μl. The plate was centrifuged at 1,000 *g* for 10 s. After 30-min incubation, 15 μl of 100× diluted furimazine in buffer was added to each well. Bioluminescence signals were recorded from both 470/40-nm and 590/35-nm channels every 1 min for a total of 30 min. The ratio (*R*) at each time point was calculated by the following equation as previously described^11^:

$$R = \frac{{T_{470\ \mathrm{nm}} - T_{590\ \mathrm{nm}} \times 0.43}}{{T_{590\ \mathrm{nm}}}},$$

where *T*_470 nm_ and *T*_590 nm_ are the total luminescent signals at 470 nm and 590 nm, respectively. For calculating the fraction of lucCageRBD dynamic range lost for serum samples in Extended Data Fig. 6, the following equation was used:

$${\mathrm{Fraction}}\,{\mathrm{of}}\,{\mathrm{lucCageRBD}}\,{\mathrm{dynamic}}\,{\mathrm{range}}\,{\mathrm{lost}} = 1 - \frac{{R_x - R_{\mathrm{min}}}}{{R_{\mathrm{max}} - R_{\mathrm{min}}}},$$

where *R*_*x*_ is the *R* with 1 nM RBD in serum sample, *R*_min_ is the *R* of serum sample but no RBD, and *R*_max_ is the *R* of 100 nM RBD in the same serum sample.

To assay 64 serum samples in Fig. 2 and Extended Data Fig. 8 (including 40 from either convalescent or vaccinated patients and 24 pre-2019 donors), 5 μl proper diluted serum sample and 5 μl of 10 nM RBD (WT or Delta) were premixed in DPBS for 20 min at room temperature. A 15-μl mixture containing 5 μl lucCageRBD (10 nM), 5 μl lucKey (10 nM) and 5 μl Antares2 (0.2 nM) was subsequently added to each well and incubated for another 10 min; 25 μl furimazine substrate (200× dilution) was added to each well, and luminescence signals were acquired immediately by Neo2 plate reader at 470/40-nm and 590/35-nm channels for a total of 20 min (1-min interval and 0.1-s exposure, and instrumental gain values were set to 120 at the 470-nm channel and 145 at the 590-nm channel). The final assay concentration contains 1 nM lucCageRBD, 1 nM lucKey, 20 pM Antares2 and 1 nM RBD. The person performing the lucCageRBD assays was blinded as to the serum samples being tested, and another person analyzed the assay data. For calculating the fraction of lucCageRBD dynamic range lost for serum samples in Fig. 2 and Extended Data Fig. 8, the following equation was used:

$${\mathrm{Fractional}}\,{\mathrm{decrease}}\,{\mathrm{in}}\,{\mathrm{lucCageRBD}}\,{\mathrm{signal}} = 1 - \frac{{R_x - R_{\mathrm{min}}}}{{R_{\mathrm{min}}}}.$$

To simplify the calculation, *R*_*x*_ here is reported as the ratio of the total luminescent signal at 470 nm over 590 nm (*T*_470 nm_/*T*_590 nm_) with 1 nM RBD in serum sample and *R*_min_ is the ratio of serum sample without the addition of RBD. Ten steady-state ratio values were averaged and assigned as *R*_*x*_ of the corresponding sample. *R*_max_ was omitted herein because *R*_max_ was unreliable with some samples due to high nAb titer.

### In vitro bioluminescence characterization of lyophilized biosensors

Five microliters of 10× lucCage and 5 μl of 10× lucKey were added to each well of a white opaque 96-well plate and lyophilized overnight. The biosensor was reconstituted in 10 μl dH_2_0 before testing, and then 84 μl of 50% HBS-EP (GE Healthcare Life Sciences) plus 50% Nano-Glo assay buffer was added to each well. The lucCage and lucKey components were incubated for 30 min at room temperature to enable pre-equilibration. One microliter of 100× diluted furimazine was added to each well. The plate was centrifuged at 1,000 *g* for 10 s. Then, 5 μl serially diluted target RBD was added to each well and measured in a A Synergy Neo2 Microplate Reader. Measurements were taken every 1 min (0.1-s integration and 10-s shaking during intervals) for a total of 90 min.

### BLI

Protein–protein interactions were measured by using an Octet RED96 System (ForteBio) using streptavidin-coated biosensors (ForteBio). Each well contained 200 μl solution, and the assay buffer was HBS-EP + buffer (GE Healthcare Life Sciences, 10 mM HEPES, pH 7.4, 150 mM NaCl, 3 mM EDTA, 0.05% (v/v) surfactant P20) plus 0.5% non-fat dry milk blotting grade blocker (Bio-Rad). The biosensor tips were loaded with analyte peptide or protein at 20 μg ml^−1^ for 300 s (threshold of 0.8-nm response), incubated in HBS-EP + buffer for 60 s to acquire the baseline measurement, dipped into the solution containing cage and/or key for 1,800 s (association step) and dipped into the HBS-EP + buffer for 1,800 s (dissociation steps). The binding data were analyzed with the ForteBio Data Analysis Software version 9.0.0.10.

### Live and pseudovirus entry and serum neutralization assays

SARS2-02 and SARS2-38 were assayed for neutralization potency by focus-reduction neutralization test as described previously^24^ and using Vero-TMPRSS2 cells. Briefly, serial dilutions of antibody were incubated with 2 × 10^2^ focus-forming units of SARS-CoV-2 of the indicated strain for 1 h at 37 °C in duplicate. Immune complexes were then added to Vero-TMRPSS2 cell monolayers in a 96-well plate and incubated for 1 h at 37 °C before the addition of 1% (w/v) methylcellulose in MEM. Following incubation for 30 h at 37 °C, cells were fixed with 4% paraformaldehyde, permeabilized and stained for infection foci with a mixture of mAbs that bind various epitopes on the RBD and NTD of spike (SARS2-02 and SARS2-38; diluted to 1 µg ml^−1^ total mAb concentration). Antibody–dose response curves were analyzed using nonlinear regression analysis (with a variable slope) (GraphPad Software).

For the mAbs CV30, B38 and CR3022 and for the vaccinated human serum samples with pseudovirus, HEK-hACE2 cells were cultured in DMEM with 10% FBS (Hyclone) and 1% PenStrep with 8% CO2 in a 37 °C incubator (Thermo Fisher Scientific). Before plating, 40 µl poly-lysine (Sigma) was placed into 96-well plates and incubated with rotation for 5 min. Poly-lysine was removed, plates were dried for 5 min then washed 1× with water before plating 2 × 10^4^ cells. The following day, cells were checked to be at 80% confluence. In a half-area 96-well plate a 1:3 serial dilution of mAb or sera was made in DMEM in 22 µl final volume. 22 µl pseudovirus was then added to the serial dilution and incubated at room temperature for 30–60 min. HEK-hACE2 plate media was removed and 40 µl of the sera/virus mixture was added to the cells and incubated for 2 h at 37 °C with 8% CO_2_. Following incubation, 40 µl 20% FBS and 2% PenStrep containing DMEM was added to the cells for 24–48 h. Following the infection, One-Glo-EX (Promega) was added to the cells in half culturing volume (40 µl added) and incubated in the dark for 5 min before reading on a BioTek plate reader. Measurements were done on all mAbs and human serum samples from each group in at least duplicate. Relative luciferase units were plotted and normalized in Prism (GraphPad) using a zero value of cells alone and a 100% value of 1:2 virus alone. Nonlinear regression of log(inhibitor) versus normalized response was used to determine IC_50_ values from curve fits.

### Sensor simulations

Mathematical models describing the ternary and quaternary sensor systems were simulated to test their responses to changes in their input parameters (concentrations and affinities of intervening species). Systems of ordinary differential equations describing the kinetics of the interactions between the species involved in each sensor were numerically integrated using integrate.odeint() as implemented in Scipy (version 1.6.3) (ref. ^25^). Steady-state values were used to determine the distribution of species at thermodynamic equilibrium.

The ternary system is composed of the following species: ACE-2, RBD, nAb, ACE-2:RBD and RBD:nAb. The following set of equations was used to describe the system:

$$\frac{{d\left[ {\mathrm{ACE2}} \right]}}{{dt}} = - k_1\left[ {\mathrm{ACE2}} \right]\left[ {\mathrm{RBD}} \right] + k_{ - 1}\left[ {\mathrm{ACE2:RBD}} \right]$$

$$\begin{array}{l}\displaystyle\frac{{d\left[ {\mathrm{RBD}} \right]}}{{dt}} = - k_1\left[ {\mathrm{ACE2}} \right]\left[ {\mathrm{RBD}} \right] + k_{ - 1}\left[ {\mathrm{ACE2:RBD}} \right]\\\qquad\qquad - k_2\left[ {\mathrm{RBD}} \right]\left[ {\mathrm{nAb}} \right] + k_{ - 2}\left[ {\mathrm{RBD:nAb}} \right]\end{array}$$

$$\frac{{d\left[ {\mathrm{nAb}} \right]}}{{dt}} = - k_2\left[ {\mathrm{RBD}} \right]\left[ {\mathrm{nAb}} \right] + k_{ - 2}\left[ {\mathrm{RBD:nAb}} \right]$$

$$\frac{{d\left[ {\mathrm{ACE2:RBD}} \right]}}{{dt}} = k_1\left[ {\mathrm{ACE2}} \right]\left[ {\mathrm{RBD}} \right] - k_{ - 1}\left[ {\mathrm{ACE2:RBD}} \right]$$

$$\frac{{d\left[ {\mathrm{RBD:nAb}} \right]}}{{dt}} = k_2\left[ {\mathrm{RBD}} \right]\left[ {\mathrm{nAb}} \right] - k_{ - 2}\left[ {\mathrm{RBD:nAb}} \right],$$

where *k*_*i*_ describe bimolecular association rate constants and *k*_-*i*_ represent unimolecular dissociation rate constants. *K*_1_ = *k*_−1_/*k*_1_ and *K*_2_ = *k*_−2_/*k*_2_ describe the equilibrium dissociation constants for the ACE-2:RBD and RBD:nAb complexes, respectively. For all ternary system simulations, *K*_1_ was set to 15 nM (ref. ^26^). For consistency with the metric used to report on the quaternary system response (fraction of lucCageRBD dynamic range lost; described in section ‘In vitro bioluminescence characterization with monoclonal antibodies’), simulations for the ternary system were reported as:

$${\mathrm{Fraction}}\,{\mathrm{sensor}}\,{\mathrm{dynamic}}\,{\mathrm{range}}\,{\mathrm{lost}} = 1 - L_{x}/L_{\mathrm{max}},$$

where *L*_*x*_ is the signal observed when all three species are present, and *L*_max_ is the response when nAb is absent.

The quaternary system is composed of the following species: closed state of the Cage-Latch, open state of the Cage-Latch (oCL), lucKey, RBD, nAb, lucKey:oCL, oCL:RBD, lucKey:oCL:RBD and RBD:nAb. Only the oCL is considered binding-competent, whereas the closed state of the Cage-Latch is not. The following set of equations was used to describe the system:

$$\frac{{d\left[ {\mathrm{cCL}} \right]}}{{dt}} = - k\left[ {\mathrm{cCL}} \right] + k_{-} \left[ {\mathrm{oCL}} \right]$$

$$\begin{array}{l}\displaystyle\frac{{d\left[ {\mathrm{oCL}} \right]}}{{dt}} = k\left[ {\mathrm{cCL}} \right] - k_{-} \left[ {\mathrm{oCL}} \right] - k_{1}\left[ {\mathrm{lucKey}} \right]\left[ {\mathrm{oCL}} \right] + k_{ - 1}\left[ {\mathrm{lucKey}}:{\mathrm{oCL}} \right]\\ - k_{2}\left[ {\mathrm{oCL}} \right]\left[ {\mathrm{RBD}} \right] + k_{ - 2}\left[ {\mathrm{oCL}}:{\mathrm{RBD}} \right]\end{array}$$

$$\begin{array}{l}\displaystyle\frac{{d\left[ {\mathrm{lucKey}} \right]}}{{dt}} = - k_{1}\left[ {\mathrm{lucKey}} \right]\left[ {\mathrm{oCL}} \right] + k_{ - 1}\left[ {\mathrm{lucKey}}:{\mathrm{oCL}} \right]\\ - k_{1}\left[ {\mathrm{lucKey}} \right]\left[ {\mathrm{oCL}}:{\mathrm{RBD}} \right] + k_{ - 1}\left[ {\mathrm{lucKey}}:{\mathrm{oCL}}:{\mathrm{RBD}} \right]\end{array}$$

$$\begin{array}{l}\displaystyle\frac{{d\left[ {\mathrm{RBD}} \right]}}{{dt}} = - k_{2}\left[ {\mathrm{oCL}} \right]\left[ {\mathrm{RBD}} \right] + k_{ - 2}\left[ {\mathrm{oCL}}:{\mathrm{RBD}} \right] - k_{2}\left[ {\mathrm{lucKey}}:{\mathrm{oCL}} \right]\left[ {\mathrm{RBD}} \right]\\ + k_{ - 2}\left[ {\mathrm{lucKey}}:{\mathrm{oCL}}:{\mathrm{RBD}} \right] - k_{3}\left[ {\mathrm{RBD}} \right]\left[ {\mathrm{nAB}} \right] + k_{ - 3}\left[ {\mathrm{RBD}}:{\mathrm{nAb}} \right]\end{array}$$

$$\frac{{d\left[ {\mathrm{nAb}} \right]}}{{dt}} = - k_{3}\left[ {\mathrm{RBD}} \right]\left[ {\mathrm{nAB}} \right] + k_{ - 3}\left[ {\mathrm{RBD}}:{\mathrm{nAb}} \right]$$

$$\begin{array}{l}\displaystyle\frac{{d\left[ {\mathrm{lucKey}}:{\mathrm{oCL}} \right]}}{{dt}} = k_{1}\left[ {\mathrm{lucKey}} \right]\left[ {\mathrm{oCL}} \right] - k_{ - 1}\left[ {\mathrm{lucKey}}:{\mathrm{oCL}} \right] \\- k_{2}\left[ {\mathrm{lucKey}}:{\mathrm{oCL}} \right]\left[ {\mathrm{RBD}} \right] + k_{ - 2}\left[ {\mathrm{lucKey}}:{\mathrm{oCL}}:{\mathrm{RBD}} \right]\end{array}$$

$$\begin{array}{l}\displaystyle\frac{{d\left[ {\mathrm{oCL}}:{\mathrm{RBD}} \right]}}{{dt}} = k_{2}\left[ {\mathrm{oCL}} \right]\left[ {\mathrm{RBD}} \right] - k_{ - 2}\left[ {\mathrm{oCL}}:{\mathrm{RBD}} \right] - k_1\left[ {\mathrm{lucKey}} \right]\left[ {\mathrm{oCL}}:{\mathrm{RBD}} \right]\\ + k_{ - 1}\left[ {\mathrm{lucKey}}:{\mathrm{oCL}}:{\mathrm{RBD}} \right]\end{array}$$

$$\begin{array}{l}\displaystyle\frac{{d\left[ {\mathrm{lucKey}}:{\mathrm{oCL}}:{\mathrm{RBD}} \right]}}{{dt}} = k_{1}\left[ {\mathrm{lucKey}} \right]\left[ {\mathrm{oCL}}:{\mathrm{RBD}} \right] - k_{ - 1}\left[ {\mathrm{lucKey}}:{\mathrm{oCL}}:{\mathrm{RBD}} \right]\\ + k_{2}\left[ {\mathrm{lucKey}}:{\mathrm{oCL}} \right]\left[ {\mathrm{RBD}} \right] - k_{ - 2}\left[ {\mathrm{lucKey}}:{\mathrm{oCL}}:{\mathrm{RBD}} \right]\end{array}$$

$$\frac{{d\left[ {\mathrm{RBD}}:{\mathrm{nAb}} \right]}}{{dt}} = k_{3}\left[ {\mathrm{RBD}} \right]\left[ {\mathrm{nAB}} \right] - k_{ - 3}\left[ {\mathrm{RBD}}:{\mathrm{nAb}} \right],$$

where *k*_*i*_ describes the bimolecular association rate constant and *k*_−*i*_ represents the unimolecular dissociation rate constant. *K*_1_ = *k*_−1_/*k*_1_, *K*_2_ = *k*_−2_/*k*_2_ and *K*_3_ = *k*_−3_/*k*_3_ describe the affinities (equilibrium dissociation constants) for the binding interfaces lucKey:oCL, oCL:RBD and RBD:nAb, respectively. The binding of lucKey and RBD to the open Cage-Latch species is considered symmetrical (i.e., noncooperative), meaning that the binding events are independent. $K = k_ {-} /k = {\mathrm{exp}}\left( {\Delta G_{\mathrm{open}}/RT} \right)$ describes the unimolecular binding equilibrium of the Latch to the Cage, with $\Delta G_{\mathrm{open}}$ the free energy of Latch opening, *R* the universal gas constant and *T* the thermodynamic temperature (set to 298.15 K for all simulations).

These systems were simulated over a range of species concentrations, as well as RBD:nAb affinities, to explore the behavior of each sensor, and gain insights into the influence of different variables on the position of the detection thresholds. The python code for running these simulations is provided as a Jupyter notebook: https://github.com/bwicky/covid_nAb_sensor_simulation.

### Statistical analysis

No statistical methods were used to predetermine the sample size. No sample was excluded from data analysis, and no blinding was used. Deidentified clinical serum samples were randomly used for spiking in target proteins. Results were successfully reproduced using different batches of pure proteins on different days. Unless otherwise indicated, data are shown as mean ± s.e.m., and error bars in figures represent s.e.m. of technical triplicates. BLI data were analyzed using ForteBio Data Analysis Software version 9.0.0.10. All data were analyzed and plotted using GraphPad Prism 8.

### Reporting Summary

Further information on research design is available in the Nature Research Reporting Summary linked to this article.

## Online content

Any methods, additional references, Nature Research reporting summaries, source data, extended data, supplementary information, acknowledgements, peer review information; details of author contributions and competing interests; and statements of data and code availability are available at 10.1038/s41587-022-01280-8.

## Supplementary information

Reporting Summary

Supplementary Table 1 The lucCageRBD assay, binding (BLI) and neutralization metrics are reported across the different monoclonal antibodies tested in Fig. 1g–o and Extended Data Fig 5a–f. Data are mean ± s.e.m.

Supplementary Table 2 The sensitivity, specificity, PPV and NPV of the lucCageRBD assay to detect if clinical serum samples at different dilutions can neutralize either SARS-CoV-2 WT or Delta VOCs.

## Data Availability

The data that support the findings of this study are available from the corresponding author upon reasonable request. All accession codes have been provided for the paper. Source data are provided with this paper.

## Source data

Source Data Fig. 1 Source data.
Source Data Fig. 2 Source data.
Source Data Extended Data Fig. 1 Source data.
Source Data Extended Data Fig. 2 Source data.
Source Data Extended Data Fig. 3 Source data.
Source Data Extended Data Fig. 4 Source data.
Source Data Extended Data Fig. 5 Source data.
Source Data Extended Data Fig. 6 Source data.
Source Data Extended Data Fig. 7 Source data.
Source Data Extended Data Fig. 8 Source data.
Source Data Extended Data Fig. 9 Source data, unprocessed gel images.
Source Data Extended Data Fig. 10 Source data.

## Code availability

The code for the sensor simulations is made available as an interactive Jupyter notebook at https://github.com/bwicky/covid_nAb_sensor_simulation. Source data are provided with this paper.
