# Supplementary material for: Thermodynamically coupled biosensors for detecting neutralizing antibodies against SARS-CoV-2 variants
Source: Nat Biotechnol. 2022 Apr 28;40(9):1336–40. doi: 10.1038/s41587-022-01280-8 (PMC9463068; doi:10.1038/s41587-022-01280-8)
Supplement: Supplementary file 1 [file 41587_2022_1280_MOESM14_ESM.pdf]

a

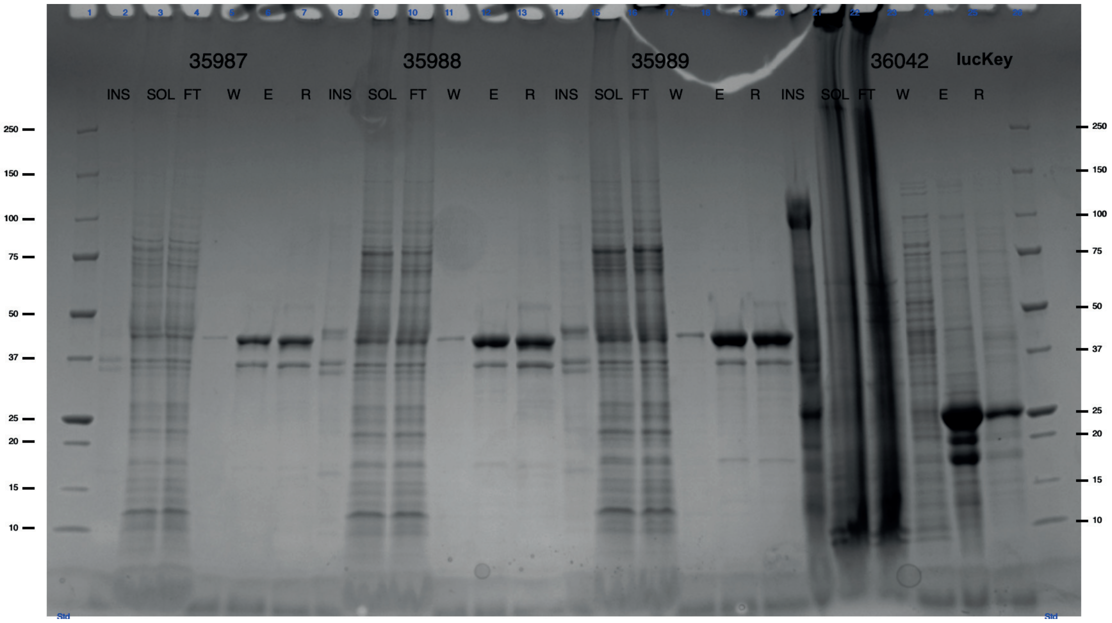
35987
35988
35989
36042
lucKey
INS SOL FT W E R INS SOL FT W E R INS SOL FT W E R INS SOL FT W E R
250
150
100
75
50
37
25
20
15
10
Std
Std

b

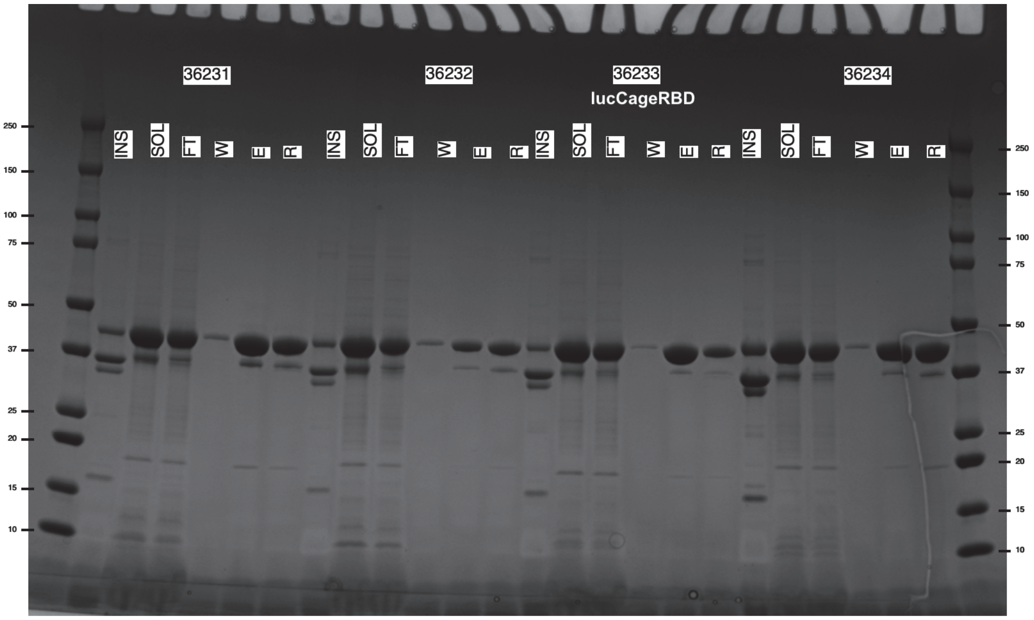

36231
36232
36233
lucCageRBD
36234
INS
SOL
FT
W
E
R
250
150
100
75
50
37
25
20
15
10

c

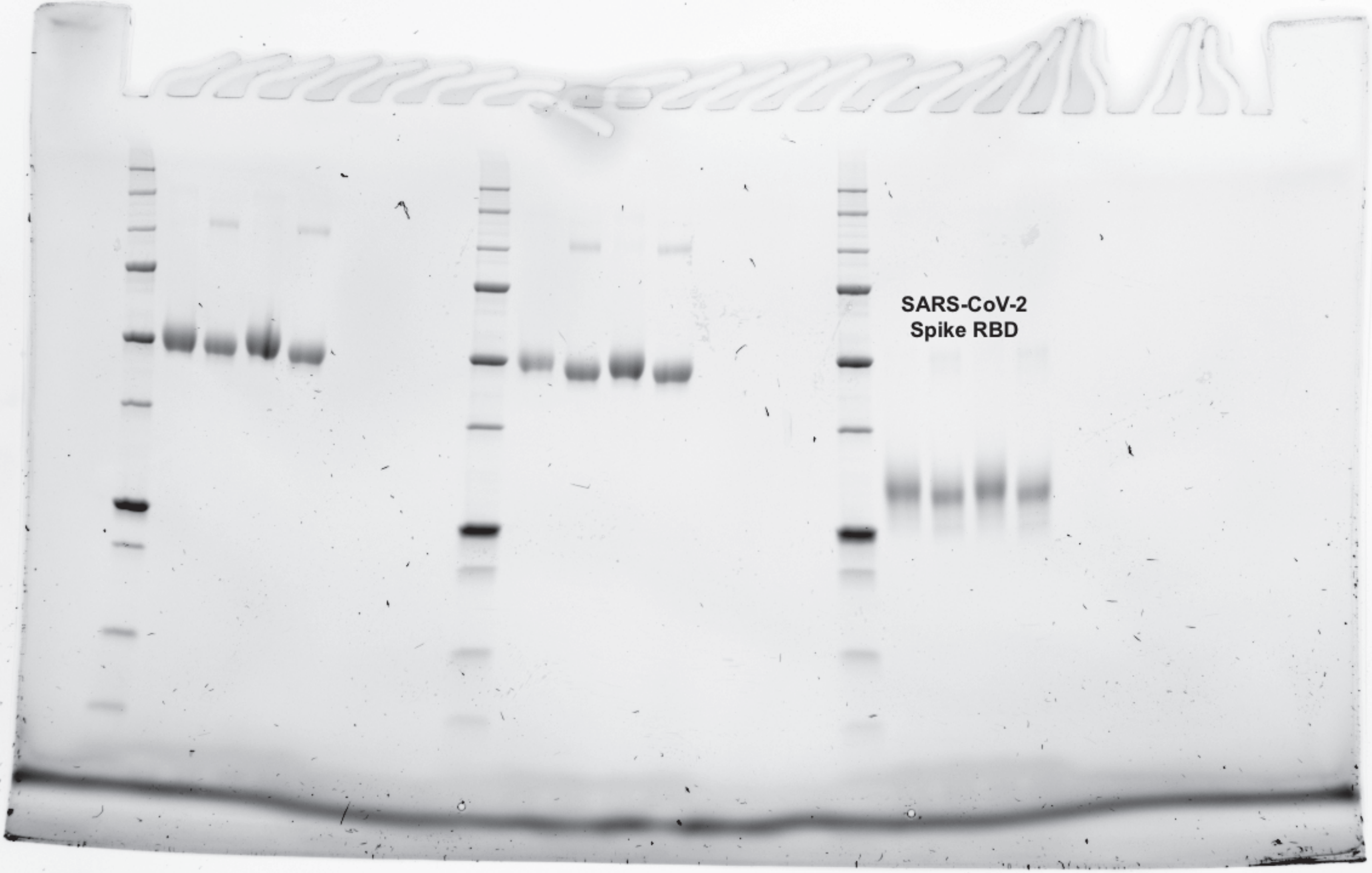

d

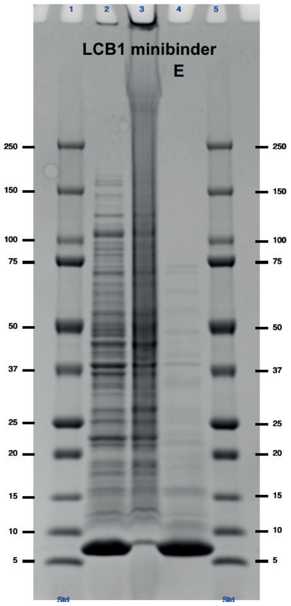

1
2
3
4
5
LCB1 minibinder
E
250
150
100
75
50
37
25
20
15
10
5
250
150
100
75
50
37
25
20
15
10
5
Std
Std

Source data for Extended Data Fig 9: Raw full gels used throughout manuscript. The raw full gels from Experimental Data Fig 9a (lucKey, left; standards, right) (a), Experimental Data Fig 9a (lucCageRBD, middle) (b), Experimental Data Fig 9b (c), and Experimental Data Fig 9c (d). INS = insoluble, SOL = soluble, FT = flow-through, W = wash, E = elution, R = residual.
