# Supplementary material for: Thermodynamically coupled biosensors for detecting neutralizing antibodies against SARS-CoV-2 variants
Source: Nat Biotechnol. 2022 Apr 28;40(9):1336–40. doi: 10.1038/s41587-022-01280-8 (PMC9463068; doi:10.1038/s41587-022-01280-8)
Supplement: Supplementary file 2 [file 41587_2022_1280_MOESM1_ESM.pdf]

nature research

Corresponding author(s): Jason Zhang, David Baker

Last updated by author(s): Nov 16, 2021

Reporting Summary

Nature Research wishes to improve the reproducibility of the work that we publish. This form provides structure for consistency and transparency in reporting. For further information on Nature Research policies, see our Editorial Policies and the Editorial Policy Checklist.

Statistics

For all statistical analyses, confirm that the following items are present in the figure legend, table legend, main text, or Methods section.

n/a	Confirmed	
☐	☒	The exact sample size (n) for each experimental group/condition, given as a discrete number and unit of measurement
☐	☒	A statement on whether measurements were taken from distinct samples or whether the same sample was measured repeatedly
☐	☒	The statistical test(s) used AND whether they are one- or two-sided<br>*Only common tests should be described solely by name; describe more complex techniques in the Methods section.*
☒	☐	A description of all covariates tested
☒	☐	A description of any assumptions or corrections, such as tests of normality and adjustment for multiple comparisons
☐	☒	A full description of the statistical parameters including central tendency (e.g. means) or other basic estimates (e.g. regression coefficient) AND variation (e.g. standard deviation) or associated estimates of uncertainty (e.g. confidence intervals)
☐	☒	For null hypothesis testing, the test statistic (e.g. F, t, r) with confidence intervals, effect sizes, degrees of freedom and P value noted<br>*Give P values as exact values whenever suitable.*
☒	☐	For Bayesian analysis, information on the choice of priors and Markov chain Monte Carlo settings
☒	☐	For hierarchical and complex designs, identification of the appropriate level for tests and full reporting of outcomes
☐	☒	Estimates of effect sizes (e.g. Cohen's d, Pearson's r), indicating how they were calculated

Our web collection on statistics for biologists contains articles on many of the points above.

Software and code

Policy information about availability of computer code

Data collection	Bioluminescence data acquired on Synergy Neo2 multi-mode microplate reader (Biotek). Biolayer interferometry data acquired on Octet RED96 (ForteBio).
Data analysis	Bioluminescnce data were analyzed and plotted using GraphPad Prism 8. Target response curves were fitted using a Sigmoidal 4PL fit in GraphPad Prism 8. BLI data was analyzed using ForteBio Data Anlaysis Software version 9.0.0.10 and plotted using GraphPad Prism 8. The code for the numerical simulations shown in this manuscript uses Scipy 1.6.3 and are available as an interactive Jupyter notebook at https://github.com/bwicky/covid_nAb_sensor_simulation.

For manuscripts utilizing custom algorithms or software that are central to the research but not yet described in published literature, software must be made available to editors and reviewers. We strongly encourage code deposition in a community repository (e.g. GitHub). See the Nature Research guidelines for submitting code & software for further information.

Data

Policy information about availability of data

All manuscripts must include a data availability statement. This statement should provide the following information, where applicable:

- Accession codes, unique identifiers, or web links for publicly available datasets
- A list of figures that have associated raw data
- A description of any restrictions on data availability

The data that support the findings of this study are available from the corresponding author upon reasonable request. All accession codes have been provided for the paper. Source data are provided with this paper. Computational source data is uploaded onto https://github.com/bwicky/covid_nAb_sensor_simulation.

Field-specific reporting

Please select the one below that is the best fit for your research. If you are not sure, read the appropriate sections before making your selection.

☒ Life sciences ☐ Behavioural & social sciences ☐ Ecological, evolutionary & environmental sciences

For a reference copy of the document with all sections, see nature.com/documents/nr-reporting-summary-flat.pdf

Life sciences study design

All studies must disclose on these points even when the disclosure is negative.

Sample size	We measured 40 SARS-CoV-2 patient samples and 30 control samples. The sample size is chosen based on the availability of patient samples and is sufficient for statistical analysis for calculating assay specificity and sensitivity.
Data exclusions	No sample was excluded from data analysis.
Replication	The results were successfully replicated using different batches of pure proteins on different days. All samples were measured at least twice and replications were successful.
Randomization	This is not relevant as this paper is an observational study, which does not involve treatment and will not be affected by relevant bias.
Blinding	Clinical samples were given with numbers that do not correlate to the characteristics of the sample. Both samples and controls were measured in the same plates under same conditions. Data was first processed in blinded mode, followed by sample/control assignment.

Reporting for specific materials, systems and methods

We require information from authors about some types of materials, experimental systems and methods used in many studies. Here, indicate whether each material, system or method listed is relevant to your study. If you are not sure if a list item applies to your research, read the appropriate section before selecting a response.

Materials & experimental systems

n/a	Involved in the study	
☐	☒	Antibodies
☐	☒	Eukaryotic cell lines
☒	☐	Palaeontology and archaeology
☒	☐	Animals and other organisms
☐	☒	Human research participants
☒	☐	Clinical data
☒	☐	Dual use research of concern

Methods

n/a	Involved in the study	
☒	☐	ChIP-seq
☒	☐	Flow cytometry
☒	☐	MRI-based neuroimaging

Antibodies

Antibodies used

1. SARS-CoV-2 antibodies SARS2-02 (produced by Diamond lab)
2. SARS-CoV-2 antibodies SARS2-38 (produced by Diamond lab)
3. SARS-CoV antibody CR3022 (produced by Baker lab)
4. SARS-CoV-2 antibodies B38 (produced by Baker lab)
5. SARS-CoV-2 antibodies CV30 (produced by Baker lab)
6. anti-V5 antibody (Thermo Fisher Scientific, 2F11F7) (dilution 1:1000)
7. anti-TMPRSS2 mAb (Abnova, Clone 2F4) (dilution 1:1000)
8. APC-conjugated goat anti-mouse IgG (BioLegend, 405308) (dilution 1:5000)

Validation

1. Validation of SARS-CoV-2 antibody SARS2-02 is described here: Liu, Z. et al. Cell Host Microbe 2021. This antibody is a murine monoclonal antibody isolated from BALB/c mice immunized with SARS-CoV-2 spike and RBD proteins. This antibody was validated to bind to the receptor binding domain of SARS-CoV-2 spike protein based on biolayer interferometry data in this work.
2. Validation of SARS-CoV-2 antibody SARS2-38 is described here: Liu, Z. et al. Cell Host Microbe 2021. This antibody is a murine monoclonal antibody isolated from BALB/c mice immunized with SARS-CoV-2 spike and RBD proteins. This antibody was validated to bind to the receptor binding domain of SARS-CoV-2 spike protein based on biolayer interferometry data in this work.
3. Validation of SARS-CoV antibody CR3022 is described here: ter Meulen, J. et al. PLoS Med 2006. Genes encoding this antibody were ordered from GenScript and cloned into pCMV/R. Antibodies were expressed by transient co-transfection of both heavy and light chain plasmids in Expi293F cells using PEI MAX (Polyscience) transfection reagent. Cell supernatants were harvested and clarified after 3 or 6 days and protein was purified using protein A chromatography (Cytiva). This antibody was validated to bind to the receptor binding domain of SARS-CoV-2 spike protein based on biolayer interferometry data in this work.
4. Validation of SARS-CoV-2 antibody B38 is described here: Wu, Y. et al. Science 2020. Genes encoding this antibody were ordered from GenScript and cloned into pCMV/R. Antibodies were expressed by transient co-transfection of both heavy and light chain

plasmids in Expi293F cells using PEI MAX (Polyscience) transfection reagent. Cell supernatants were harvested and clarified after 3 or 6 days and protein was purified using protein A chromatography (Cytiva). This antibody was validated to bind to the receptor binding domain of SARS-CoV-2 spike protein based on biolayer interferometry data in this work.
5. Validation of SARS-CoV-2 antibody CV30 is described here: Hurlburt, N.K. et al. Nat Commun 2020. Genes encoding this antibody were ordered from GenScript and cloned into pCMV/R. Antibodies were expressed by transient co-transfection of both heavy and light chain plasmids in Expi293F cells using PEI MAX (Polyscience) transfection reagent. Cell supernatants were harvested and clarified after 3 or 6 days and protein was purified using protein A chromatography (Cytiva). This antibody was validated to bind to the receptor binding domain of SARS-CoV-2 spike protein based on biolayer interferometry data in this work.
6. According to manufacturer, sequenced and validated via immunoblotting, immunostaining, and immunoprecipitation.
7. According to manufacturer, sequenced and validated via ELISA.
8. According to manufacturer, this polyclonal goat anti-mouse IgG antibody has no cross-reactivity against non-immunoglobulin serum proteins. Each lot of this antibody is quality control tested by immunofluorescent staining with flow cytometric analysis.

Eukaryotic cell lines

Policy information about cell lines

Cell line source(s)	HEK293T/17 (ATCC, CRL-11268). HEK-ACE2 (BEI Resources, NR-52511). HEK293F (Invitrogen, K9000-01). Epi293F (Life Technologies, A14527). Vero E6 (ATCC, CRL-1586). Vero-TMPRSS2 (Siyuan Ding lab). Vero-hACE2-TMPRSS2 (Barney Graham lab).
Authentication	Cells were not further authenticated in the laboratory.
Mycoplasma contamination	All cells were tested negative for Mycoplasma by the provider, and it was not further confirmed in the laboratory.
Commonly misidentified lines (See ICLAC register)	No commonly misidentified lines were used in this study.

Human research participants

Policy information about studies involving human research participants

Population characteristics	Serum specimens were derived from excess plasma or sera from adults (>18yo) of both genders kindly provided by the Director of the Clinical Chemistry Division, the hospital of University of Washington. All anonymized donor specimens were provided de-identified.
Recruitment	The donors consented to have their excess specimens be used for other experimental studies, they could be transferred to our study without additional consent.
Ethics oversight	The Clinical Chemistry Division, the hospital of University of Washington, with the approval by the University of Washington Human Subjects Division

Note that full information on the approval of the study protocol must also be provided in the manuscript.
